# A Novel Multiple Instance Learning Method Based on Extreme Learning Machine

**DOI:** 10.1155/2015/405890

**Published:** 2015-02-03

**Authors:** Jie Wang, Liangjian Cai, Jinzhu Peng, Yuheng Jia

**Affiliations:** School of Electrical Engineering, Zhengzhou University, Zhengzhou 450001, China

## Abstract

Since real-world data sets usually contain large instances, it is meaningful to develop efficient and effective multiple instance learning (MIL) algorithm. As a learning paradigm, MIL is different from traditional supervised learning that handles the classification of bags comprising unlabeled instances. In this paper, a novel efficient method based on extreme learning machine (ELM) is proposed to address MIL problem. First, the most qualified instance is selected in each bag through a single hidden layer feedforward network (SLFN) whose input and output weights are both initialed randomly, and the single selected instance is used to represent every bag. Second, the modified ELM model is trained by using the selected instances to update the output weights. Experiments on several benchmark data sets and multiple instance regression data sets show that the ELM-MIL achieves good performance; moreover, it runs several times or even hundreds of times faster than other similar MIL algorithms.

## 1. Introduction

Multiple instance learning (MIL) was first developed to solve the problem of drug prediction [1]. From then on, a variety of problems are formulated as multiple instance ones, such as object detection [2], image retrieval [3], computer aided diagnosis [4], visual tracking [5–7], text categorization [8–10], and image categorization [11, 12]. In MIL, the single example object that is called a bag contains many feature vectors (instances), some of which may be responsible for the observed classification of the example or object, and the label is only attached to bags (training examples) instead of its instances. Furthermore, example is classified as positive if at least one of its instances is a positive example; otherwise, the bag is labeled as a negative one.

Numerous learning methods for MIL problem have been proposed in the past decade. As the first learning algorithm for MIL, Axis-Parallel Rectangle (APR) [1] was created by changing a hyper rectangle in the instances feature space. Then, the famous Diverse Density (DD) [13] algorithm was proposed to measure a cooccurrence of similar instances from different positive bags. Andrews et al. [8] used support vector machine (SVM) to solve the MIL problem that was called MI-SVM, where a maximal margin hyperplane is chosen for the bags by regarding a margin of the most positive instance in a bag. Wang and Zucker [14] proposed two variants of the *k*-nearest neighbor algorithm by taking advantage of the *k*-neighbors at both the instance and the bag, namely, Bayesian-*k*NN and Citation-*k*NN. Chevaleyre and Zucker derived ID3-MI [15] for multiple instances learning from the decision tree algorithm ID3. The key techniques of the algorithm are the so-called a multiple instance coverage and a multiple instance entropy. Zhou and Zhang presented a multiple instance neural network named BP-MIL [16] with a global error function defined at the level of bags. Nevertheless, it is not uncommon to see that it takes a long time to train most of the multiple instance learning algorithms.

Extreme learning machine (ELM) provides a powerful way for learning pattern which has several advantages such as faster learning speed, higher generalization performance [17–19]. This paper is mainly concerned with extending extreme learning machine to multiple instance learning. In this paper, a novel classification method based on neural network is presented to address MIL problem. Two-step training procedure is employed to train the ELM-MIL. During the first step, the most qualified instance is selected in each bag through SLFNs with a global error function defined at the level of bags, and the single selected instance is used to represent each bag. During the second step, by making use of the selected instances, the modified SLFNs output parameters are optimized the way ELM does. Experiments on several benchmark data sets and text categorization data sets show that the ELM-MIL achieves good performance; moreover, it runs several times or even hundreds of times faster than other similar MIL algorithms.

The remainder of this paper is organized as follows. In Section 2, ELM is briefly introduced and an algorithmic view of the ELM-MIL is provided. In Section 3, the experiments on various MIL problems are conducted and the results are reported. In Section 4, the main idea of the method is concluded and possible future work is discussed.

## 2. Proposed Methods

In this section, we first introduce ELM theory; then, a modified ELM is proposed to address the MIL problem, where the most positive instance in positive bag or the least negative instance in negative bag is selected.

### 2.1. Extreme Learning Machine

ELM is a single hidden layer feedforward neural network where the hidden node parameters (e.g., the input weights and hidden node biases in additive nodes and Fourier series nodes, centers, and impact factors in RBF nodes) are chosen randomly and the output weights are usually determined analytically by using the least square method. Because updating of the input weights is unnecessary, the ELM can learn much faster than back propagation (BP) algorithm [18]. Also, ELM can achieve a better generalization performance.

Concretely, suppose that we are given a training set comprising *N* samples {**x**_*i*_, *y*_*i*_}_*i*=1_^*N*^ and the hidden layer output (with *L* nodes) denoted as a row vector **o**(**x**) = [*o*_1_(**x**),…, *o*_*L*_(**x**)], where **x** is the input sample. The model of the single hidden layer neural network can be written as

(1) $$\begin{array}{c} o_{i}=\overset{L}{\underset{j=1}{\sum }}\beta_{j}G\left(a_{j},b_{j},x_{i}\right)\;i=1,2,\ldots ,N, \end{array}$$

where **β**_*j*_ is the weight of *j*th hidden node connecting to output node, *o*_*i*_ is the output of the network with *L* hidden nodes, and **a**_*j*_ and *b*_*j*_ are the input weights and hidden layer bias, respectively. *G*(**a**_*j*_, *b*_*j*_, **x**_*i*_) is the hidden layer function or kernels. According to the ELM theory [18–20], the parameters **a**_*i*_ and *b*_*i*_ can be randomly assigned, and the hidden layer function can be a nonlinear continuous function that satisfies universal approximation capability theorems. In general, the popular mapping functions are as follows:

(1) Sigmoid function:
  (2) $$\begin{array}{c} G\left(a,b,x\right)=\frac{1}{1+\exp\left(-ax+b\right)}, \end{array}$$
(2) Gaussian function:
  (3) $$\begin{array}{c} G\left(a,b,x\right)=\exp\left(-b{\left\|x-a\right\|}^{2}\right). \end{array}$$

For notational simplicity, (1) can be written as

(4) $$\begin{array}{c} O=H\beta , \end{array}$$

where **H** is the *N* × *L* hidden layer output matrix, whose elements are as follows:

(5) $$\begin{array}{c} H=\left[\begin{array}{ccc} G\left(x_{1},a_{1},b_{1}\right) & \cdots & G\left(x_{1},a_{L},b_{L}\right)\\ \vdots & \cdots & \vdots \\ G\left(x_{N},a_{1},b_{1}\right) & \cdots & G\left(x_{M},a_{L},b_{L}\right) \end{array}\right] \end{array}$$

and **o**(**x**) = [*o*_1_(**x**),…, *o*_*N*_(**x**)] and **β** = [**β**_1_,…, **β**_*L*_].

The least square solution with minimal norm is analytically determined by using generalized Moore-Penrose inverse:

(6) $$\begin{array}{c} \beta =H^{†}Y, \end{array}$$

where **H** ^†^ is the Moore-penrose generalized inverse of the hidden layer output matrix **H**.

### 2.2. ELM-MIL

Assume that the training set contains *M* bags, the *i*th bag is composed of *N*_*i*_ instances, and all instances belong to the *p*-dimension space; for example, the *j*th instance in the *i*th bag is [*B*_*ij*1_, *B*_*ij*2_,…, *B*_*ijp*_]. Each bag is attached by a label *Y*_*i*_. If the bag is positive, then *Y*_*i*_ = 1; otherwise, *Y*_*i*_ = 0. Our goal is to predict whether the label of new bags is positive or negative. Hence, the global error function is defined at the level of bags instead of at the level of instances:

(7) $$\begin{array}{c} E=\overset{N}{\underset{i=1}{\sum }}E_{i}, \end{array}$$

where *E*_*i*_ is the error on bag *B*_*i*_.

Based on the assumption if a bag is positive at least one of its instances is positive, we can simply define *E*_*i*_ as follows:

(8) $$\begin{array}{c} E_{i}=\frac{1}{2}{\left(\underset{1\leq j\leq N_{i}}{\max}\left(o_{ij}\right)-Y_{i}\right)}^{2}, \end{array}$$

where *o*_*ij*_ is the output of instance for bag *B*_*i*_. And our goal is to minimize the cost function for the bags.

Up to now, the last problem is how we can find the most likely instance that has the maximum output. As we know, ELM chooses the input weights randomly and determines the output weights of SLFNs analytically. At first, the output weights are not known; thus, the max⁡_1≤*j*≤*N*_*i*__(*o*_*ij*_) can not be calculated directly [16]. Furthermore, both the input weights/hidden node biases and output weights are initialized randomly. When the bags are put into the original SLFNs one by one, the instance having the maximum output will be marked down. The most positive or least negative instance (having maximum output) will be thus picked out from each bag. For each bag, we pick the most positive or negative instance with highest likelihood according to the label of the bags. The selected instances, whose number is equal to the number of training bags, will be used as training data set to train the original network through minimizing the least square.

Given a training set {*B*_*i*_, *Y*_*i*_∣*i* = 1,…, *M*}, the bag *B*_*i*_ containing *N*_*i*_ instances {*B*_*i*1_, *B*_*i*2_,…, *B*_*iN*_*i*__}, each instance is denoted as *p*-dimension feature vector, so the *j*th instance of the *i*th bag is [*B*_*ij*1_, *B*_*ij*2_,…, *B*_*ijp*_]^*T*^. The hidden node uses sigmoid function, and hidden node number is defined as *L*. The algorithm can now be summarized step-by-step as follows.

**Step 1 .**

Randomly assign the input weight **α** = [**α**_1_,…, **α**_*L*_], the bias **b** = [*b*_1_,…, *b*_*L*_] and output weight **β** = [**β**_1_,…, **β**_*L*_], respectively.

**Step 2 .**

**For** every bag *B*_*i*_

     ** For** every instance *B*_*ij*_ in bag *B*_*i*_

      Calculate the output of the SLFNs *o*_*ij*_:

(9) $$\begin{array}{c} o_{ij}=H\left(B_{ij},\alpha ,b\right)\beta , \end{array}$$

where **H**(*B*_*ij*_, **α**, **b**) = [*G*(*B*_*ij*_, **α**_1_, *b*_1_),…, *G*(*B*_*ij*_, **α**_*L*_, *b*_*L*_)] and *G*(*B*_*ij*_, **α**, *b*) is the output of the hidden node function; here the sigmoid function equation (2) is used.

     ** End for**

    Select the win-instance *B*_*i*_win_:

(10) $$\begin{array}{c} B_{i\_\text{win}}=\underset{1\leq j\leq N_{i}}{\arg\;\max}\left(o_{ij}\right). \end{array}$$

    ** End for**

Now, we have *M* win-instances as the model input *B*_win_ = {*B*_1_win_, *B*_2_win_,…, *B*_*i*_win_,…, *B*_*M*_win_}.

**Step 3 .**

Calculate the hidden layer output matrix **H**:

(11) $$\begin{array}{c} H=\left[\begin{array}{ccc} G\left(B_{1\_\text{win}},\alpha_{1},b_{1}\right) & \cdots & G\left(B_{1\_\text{win}}\cdot \alpha_{L},b_{L}\right)\\ \vdots & \cdots & \vdots \\ G\left(B_{M\_\text{win}},\alpha_{1},b_{1}\right) & \cdots & G\left(B_{M\_\text{win}}\cdot \alpha_{L},b_{L}\right) \end{array}\right]. \end{array}$$

**Step 4 .**

Calculate the new output weights:

(12) when  N<L: β=H†Y=HTIC+HHT−1Ywhen  N>L: β=H†Y=IC+HTHHTY,

where **Y** = [*Y*_1_, *Y*_2_,…, *Y*_*M*_], **H** ^†^ is the Moore-penrose generalized inverse of the hidden layer output matrix **H**, and 1/*C* is a regulator parameter added to the diagonal of **H** ^*T*^ **H** for achieving better generalization performance.

## 3. Experiments

### 3.1. Benchmark Data Sets

Five most popular benchmark MIL data sets are used to demonstrate the performances of the proposed methods, which are the MUSK1, MUSK2, and images of Fox, Tiger, and Elephant [21]. The data sets MUSK1 and MUSK2 consist of descriptions of molecules (bags). MUSK1 has 92 bags of which 47 bags are labeled as positive and the other are negative. MUSK2 has 102 bags of which 39 bags are labeled as positive bags and the other are negative. The number of instances in each bag in MUSK1 is 6 on average, while in MUSK2 the number is more than 60 on average. And the instance in MUSK data sets is defined by a 166-demensional feature vector. For Fox, Tiger, and Elephant data sets from image categorization, each of them contains 100 positive and 100 negative bags, and each instance is a 230-dimensional vector. The main goal is to differentiate images containing elephants, tigers and foxes from those that do not, respectively. More information of the data sets can be found in [8].

ELM-MIL network with 166 input units, where each unit corresponds to a dimension of the feature vectors, is trained for ranging hidden units. It should be noted that outputs [0.5,1] are positive for each unit output, while [0,0.5] are negative. When applied for multiple instance classification, our method involves two parameters, namely, the regular parameter *C* and the number of hidden neurons. In the experiments, *C* and the number of hidden neurons are selected from {2^−3^, 2^−2^, 2^−1^, 2^0^, 2^1^, 2^2^, 2^3^, 2^4^, 2^5^, 2^6^, 2^7^} and {10,50,100,150,200,250,300,350,400,450,500,550,600}, respectively. For comparison with several typical MIL methods, we conduct 10-fold cross validation, which is further repeated 10 times with random different partitions, and the average test accuracy is reported. In Table 1, our method is compared with iterated-discrim APR, Diverse Density, EM-DD, BP-MIP, MI-SVM, C4.5, and Citation-*k*NN. All the results taken from original literature were obtained via 10-fold cross validation (10CV) except Citation-*k*NN using leaving one out cross validation (LOO). The values in bracket are the standard deviation and the unavailable results are marked by N/A.

**Figure 1:**
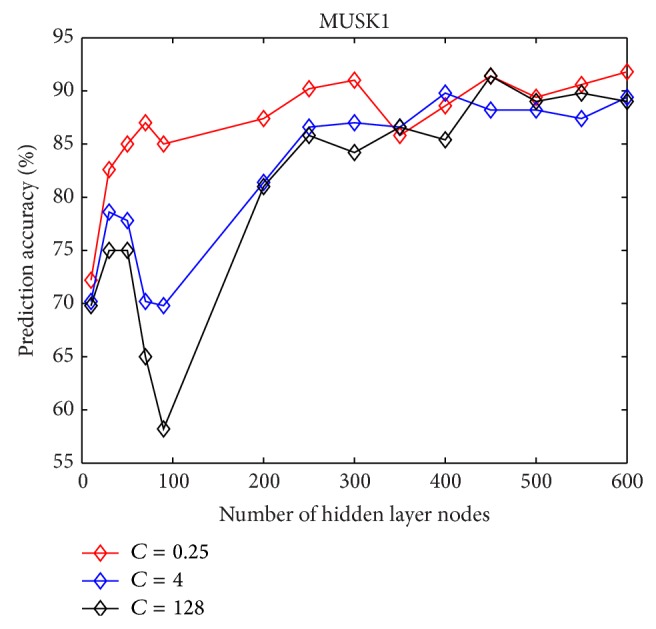
The predictive accuracy of MI-ELM on MUSK1 changes as the number of hidden neurons increases.

**Figure 2:**
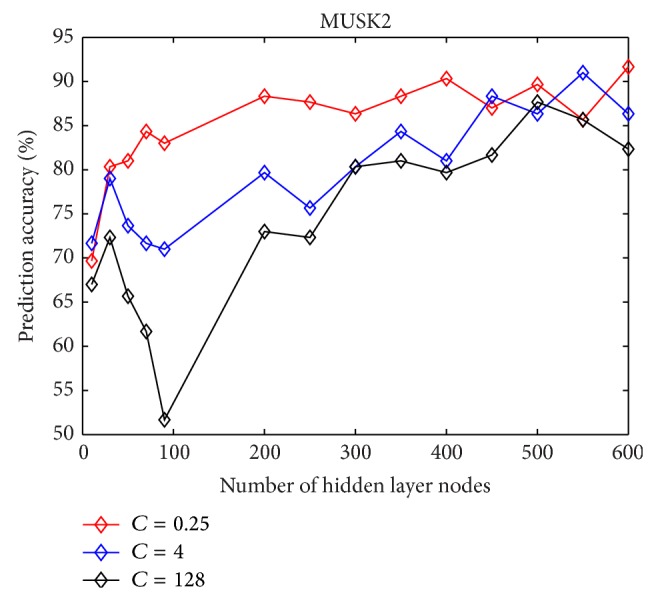
The predictive accuracy of MI-ELM on MUSK2 changes as the number of hidden neurons increases.

The relation between the number of hidden layer nodes and the prediction accuracy with different regulator parameter *C* on MUSK1 and MUSK2 data sets is presented in Figures 1 and 2, respectively. It can be found that when the number of hidden layer is over 300, the accuracy stays at a high level for both MUSK1 and MUSK2.

As time is limited, we have conducted experiments on several typical algorithms and recorded their computation time. The training of ELM-MIL, Citation-*k*NN, BP-MIP, and Diverse Density method are all executed on a 2.6 GHz, i5-3230 PC, matlab2013b. Since Citation-*k*NN, Diverse Density, and BP-MIP are all time-consuming algorithms, the time recorded below is based on the total training time of 10CV instead of LOO. The results are shown in Table 2 for MUSK1 and Table 3 for MUSK2.

Table 1 suggests that MI-ELM is comparable with state-of-the-art algorithm that is proposed in [13]. Particularly, it can be found from Tables 2 and 3 that the test accuracy of ELM-MIL not only is higher than that of BP-MIP, which is also a multiple instance learning method based on neural network, but also learns significantly faster than that of BP-MIP on MUSK data set. Moreover, the iterated-discrim APR algorithm was specially devised for MUSK data, while ELM-MIL is a general algorithm. It is clear that, for applicability, ELM-MIL is superior to the APR method. When compared with Citation-*k*NN, from the point of prediction accuracy, ELM-MIL is worse than Citation-*k*NN, but from the point of learning time (see Tables 2 and 3) ELM-MIL runs several times faster than Citation-*k*NN. In addition, ELM-ELM has some advantage compared with other MIL algorithms like Diverse Density, MI-kernel, EM-DD, MI-SVM, C4.5. For example, from Tables 1, 2, and 3, it can be seen that ELM-MIL runs hundreds of times faster than Diverse Density and its performance is also comparable on both Musk1 and Musk2. In addition, both Lozano-Perez's Diverse Density and EM-DD employed some feature selection. Since EM-DD and MI-kernel have a mount of parameters to set, it is reasonable to infer that their learning speed is very slow compared with ELM-MIL.

### 3.2. Multiple Instance Regression

We compare ELM-MIL, BP-MIP, Diverse Density, and MI-kernel [22] on several multiple instance regression data sets, which are named as LJ-*r*.*f*.*s*. As for LJ-*r*.*f*.*s*, *r* is the number of relevant features, *f* is the number of features, and *s* is the number of scale factors used for the relevant features indicating the importance of the features. The suffix S suggests that, to partially mimic the MUSK data set, the data set uses only labels that are not near 1/2. Each data set is composed of 92 bags. More information for the regression data sets can be found in [23]. Here four data sets are used, including LJ-160.166.1, LJ160.166.1-S, LJ-80.166.1, and LJ-80.166.1-S. And we also perform 10CV tests and report the square loss as well as the computation time in Table 4. Note that the table shows the 10CV results reported in literature, including BP-MIP, Diverse Density, and MI-kernel. All of them run on a 2.6 GHz, i5-3230 PC, matlab2013b. Table 4 shows that the square loss of our proposed ELM-MIL is worse than MI-kernel, but ELM-MIL takes only tiny mounts of seconds to find appropriate parameters, about twenty times faster than MI-kernel. When compared with BP-MIP and Diverse Density, from the point of performance as well as from the point of training time, ELM-MIL is better than both of them. These results indicate that ELM-MIL is an efficient and effective approach on multiple instance regression task.

## 4. Conclusions

In this paper, a novel multiple instance learning algorithm is proposed based on extreme learning machine. Through modifying the specific error function for the characteristics of multiple instance problems, the most representative instance is chosen in each bag, and the chosen instances are employed to train the extreme learning machine. We have tested ELM-MIL over the benchmark data sets which are taken from applications of drug activity prediction, artificial data sets, and multiple instance regression. Compared with other methods, ELM-MIL algorithm learns much faster and its classification accuracy is slightly worse than state-of-the-art multiple instance algorithms. The experimental results recorded in this paper are rather preliminary. For continuous work, there may be two directions. First, it is possible to improve our method performance by exploiting feature selection techniques [3, 13], that is, feature scaling with Diverse Density and feature reduction with principal component analysis. Next, one can build ensembles of several multiple instance learners to enhance the basic multiple instance learners.

## Figures and Tables

**Table 1:** ELM-MIL performance on benchmark data sets.

Algorithm	MUSK1	MUSK2	Elephant	Fox	Tiger
Iterated-discrim APR [1]	92.4	89.2	N/A	N/A	N/A
Citation-*k*NN [14]	92.4	86.3	N/A	N/A	N/A
Diverse Density [13]	88	84	N/A	N/A	N/A
ELM-MIL (proposed)	86.5 (4.2)	85.8 (4.6)	76.7 (3.9)	59.5 (3.7)	74.6 (2.4)
EM-DD [3]	84.8	84.9	78.3	56.1	72.4
BP-MIP [16]	83.7	80.4	N/A	N/A	N/A
MI-SVM [8]	77.9	84.3	81.4	59.4	84
C4.5 [15]	68.5	58.5	N/A	N/A	N/A

**Table 2:** Accuracy and computation time on MUSK1.

Algorithm	Accuracy	Computation time (min)
ELM-MIL	86.5	**0.12**
Citation-*k*NN	**92.4**	1.1
BP-MIP	83.8	110
Diverse Density	88	350

**Table 3:** Accuracy and computation time on MUSK2.

Algorithm	Accuracy	Computation time (min)
ELM-MIL	85.8	**18**
Citation-*k*NN	**86.3**	140
BP-MIL	84	1200
Diverse Density	84	3600

**Table 4:** Squared loss and computation time (second) on regression data sets.

Squared loss Training time	LJ-160.166.1		LJ-160.166.1-S		LJ-80.166.1		LJ-80.166.1-S	
MI-Kernel	**0.00116**	90	**0.0127**	8000	**0.0174**	120	**0.0219**	10100
ELM-MIL	0.0376	**5.3**	0.0648	**45.6**	0.0485	**6.8**	0.0748	**42.7**
BP-MIP	0.0398	4980	0.0731	13000	0.0487	5100	0.0752	12500
Diverse Density	0.0852	12000	0.0052	17000	N/A	N/A	0.1116	17600
